# Ciprofloxacin-Coated Tympanostomy Tubes with Sustained-Release Varnish: A Novel Strategy to Combat Biofilm Formation by *Pseudomonas aeruginosa*

**DOI:** 10.3390/microorganisms13092039

**Published:** 2025-08-31

**Authors:** Sari Risheq, Andres Sancho, Michael Friedman, Irith Gati, Ron Eliashar, Doron Steinberg, Menachem Gross

**Affiliations:** 1 Department of Otolaryngology-Head and Neck Surgery, Hadassah Medical Center, Jerusalem 9112102, Israelgross@hadassah.org.il (M.G.); 2 The Biofilm Research Laboratory, Faculty of Dental Medicine, Hebrew University of Jerusalem, Jerusalem 9190500, Israel; dorons@ekmd.huji.ac.il; 3 Institute for Drug Research, School of Pharmacy, Hebrew University of Jerusalem, Jerusalem 9190500, Israel; 4 Faculty of Medicine, Hebrew University of Jerusalem, Jerusalem 9190500, Israel

**Keywords:** otitis media with effusion (OME), tympanostomy tubes (TTs), ciprofloxacin, sustained-release varnish (SRV), biofilm formation, *Pseudomonas aeruginosa*, postoperative otorrhea, localized antibiotic delivery, otolaryngology

## Abstract

Objective: The aim of this study is to develop and evaluate the antibacterial and anti-biofilm efficacy of ciprofloxacin-coated tympanostomy tubes (TTs) using a sustained-release varnish (SRV-CIPRO) and introduce a novel tympanic membrane model for preclinical evaluation. Study Design: This was an in vitro experimental study. Setting: This study was conducted in a biofilm research laboratory in an academic medical center. Methods: Sterile fluoroplastic TTs were coated with SRV-CIPRO or placebo varnish. A novel tympanic membrane (TM) model was developed using a layered agar–plastic system. Antibacterial activity, biofilm inhibition, and bacterial viability were assessed through agar diffusion, MTT, ATP quantification, HR-SEM, and SD-CLSM. Results: SRV-CIPRO-coated TTs exhibited sustained antibacterial activity for up to 10 days. Compared to the placebo, SRV-CIPRO significantly inhibited biofilm formation, reduced metabolic activity, and decreased bacterial viability (*p* < 0.05). Imaging confirmed fewer bacterial colonies on SRV-CIPRO TTs. The TM model allowed realistic testing of tube insertion and infection simulation. Conclusion: SRV-CIPRO-coated TTs offer sustained antibiotic delivery, potentially reducing postoperative otorrhea and biofilm-related complications. The TM model provides a platform for preclinical evaluation of middle ear devices.

## 1. Introduction

Otitis media with effusion (OME) is a common condition affecting the middle ear, particularly in children. It is a leading cause of conductive hearing loss and contributes to developmental and speech delays, resulting in poor school performance and developmental challenges [1,2,3,4,5]. An estimated 90% of preschool children experience OME once in their life [6]. Early diagnosis of OME is essential to preventing complications that could result in more severe conditions such as tympanosclerosis, tympanic membrane retraction, and cholesteatoma due to the retraction of the pocket of the tympanic membrane [5,7]. Initial early diagnosis of OME and treatment with tympanostomy tubes (TTs) is essential [8]. TT insertion, also known as ventilation tube or grommet insertion, is a widely used surgical procedure to treat chronic OME or recurrent acute otitis media (AOM) in children [9,10], and occasionally in adolescents and adults [11]. The procedure involves placing the tube in the tympanic membrane to ventilate the middle ear, preventing fluid accumulation, and thereby reducing the risk of further infections or complications [12]. Typically, the TTs remain in place for 6 to 18 months, and are naturally expelled [13,14]. The most common complication is TT otorrhea (TTO), occurring in up to 26% of children [15]. TTO is divided into immediate (early), postoperative otorrhea (within 4 weeks) and delayed otorrhea (more than 4 weeks postoperatively) [16]. The bacteria isolated from patients with TTO is mainly *Pseudomonas aeruginosa* (*P. aeruginosa*) and occasionally *Staphylococcus aureus* (*S. aureus*).

Ciprofloxacin, a broad-spectrum fluoroquinolone antibiotic, in ear-drop form is commonly used as prophylactic treatment following the insertion of TTs to prevent early TTO [17,18]. Ciprofloxacin is highly effective against common bacterial pathogens such as *P. aeruginosa* [19] and *S. aureus* [20]. Topical application of ciprofloxacin ensures that high concentrations of the antibiotic directly reach the infection site without the systemic side effects associated with oral antibiotics, making it a preferred drug of choice for postoperative care [21]. Additionally, the absence of ototoxicity with topical ciprofloxacin makes it a preferred option for prophylaxis in patients undergoing TT surgery [22,23,24]. In cases of chronic ear discharge from TTs, the bacterial biofilm on the tube prevents antibiotic drops from reaching and acting against bacteria on the TTs.

Sustained-release delivery systems are technologies that allow a controlled and prolonged release of a drug. Varnishes are liquid pharmaceutical preparations that are applied to a surface and when they are dry, leave a thin film coating. Sustained-release varnish (SRV) allows for a controlled release rate of the drug, allowing for longer term effects. The general pharmaceutical formulation of a varnish consists of polymers, pharmaceutical release controllers, and active agents, dissolved or mixed in a suitable solvent. Various types of SRVs have been proposed and tested [25]. The advantages of sustained-release devices are the controlled amount of the drug to be released in the desired location, avoiding systemic delivery of the drug, minimization of dosages, fewer side effects, and finally, higher patient compliance.

In this study, we have developed a novel technology for coating TTs with an SRV containing ciprofloxacin (SRV-CIPRO) to abrogate biofilm formation on both the TTs and the surrounding environment, and evaluated the effect of this combination using an innovative tympanic membrane model for TT insertion.

## 2. Materials and Methods

### 2.1. Preparation of Sustained-Release Varnish (SRV)

The SRV containing ciprofloxacin (SRV-CIPRO) was formulated using a modified method based on Friedman et al. [26]. Ethylcellulose (Ethocel N-100, Hercules Inc., Wilmington, DE, USA) and polyethylene glycol 400 (PEG 400, Sigma-Aldrich, Saint Louis, MO, USA) formed the polymeric matrix, while CIPRO served as the active antimicrobial agent. Ethanol was utilized as the solvent, with additional pharmaceutical excipients, such as Klucel, PEG, and hydroxypropyl cellulose, enhancing adhesion to smooth surfaces and controlling the release rate. The placebo varnish (SRV-placebo) followed the same preparation procedure above, excluding ciprofloxacin.

### 2.2. Tympanostomy Tubes (TTs)

**Figure 1:**
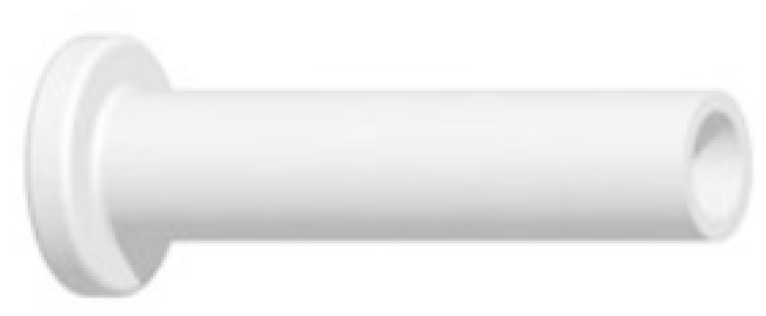
Tympanostomy tube.

Various sizes of tympanostomy tubes are available on the market; however, for this study, sterile fluoroplastic tubes (GRACE Medical, Memphis, TN, USA), with an inner diameter of 1.14 mm, and uniform shape and weight, were used (Figure 1). These tubes ensured consistency across all experiments, minimizing variability and enhancing the reliability of the experimental results.

### 2.3. Coating of Tympanostomy Tubes

Sterile TTs were coated via the dipping coat immersion method, either with the SRV-CIPRO or SRV-placebo formulations. After the immersion procedure, the coated tubes were air-dried at room temperature for 24 h. This process was repeated three times to ensure consistent coating. The amount of SRV-CIPRO applied was quantified by weighing the tubes before and after coating. This study compared the antimicrobial efficacy of SRV-CIPRO versus SRV-placebo.

### 2.4. Bacterial Strain

The bacterial strain *Pseudomonas aeruginosa* PAO1 was used as the model organism for biofilm formation. The bacteria were obtained from the −80 °C stocks of Prof. Steinberg’s laboratory. Bacterial cultures were grown overnight at 37 °C in tryptic soy broth (TSB) (Acumedia, Neogen, Lansing, MI, USA), with fresh cultures prepared daily for each experiment. The bacterial concentration was standardized to the same optical density (OD_600nm_) for all studies to ensure consistency.

### 2.5. Biofilm Model

TTs coated with SRV-CIPRO or SRV-placebo were placed in test tubes containing 3 mL of TSB supplemented with 1% glucose (TSBG) and inoculated with 30 µL of overnight standardized *P. aeruginosa* culture. The tubes were incubated for 24 h at 37 °C, followed by daily transfers to fresh bacterial cultures in TSBG.

### 2.6. Tympanic Membrane Model for TTs Insertion

**Figure 2:**
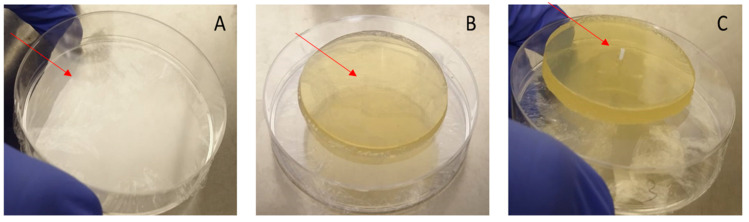
Preparation of the tympanic membrane model. (**A**) Plate with plastic roll. (**B**) TSA above the plate. (**C**) Final model with the insertion of the tympanostomy tube.

We aimed to develop a model of the tympanic membrane (TM) mimicking the structural and functional properties of the human TM. This model serves as a platform for evaluating the insertion and performance of TTs under conditions simulating the human middle ear environment. To construct the model, we utilized a round sterile plate as the base, over which a layer of transparent plastic roll was applied. The plastic roll, sterilized with ethanol, serves as the foundational layer that mimicked a human TM’s flexibility and translucency. To further replicate the biological structure, we covered the plastic roll with a thin layer of Tryptic Soy Agar (TSA) cut into a round or oval shape, resembling the shape and dimensions of the human TM. Fresh bacteria were then added to the model. A small incision was then made in both layers (the TSA agar and the plastic roll) to create an opening for the insertion of the TM (Figure 2). The tube was inserted in an upright orientation, simulating the positioning of a TT inside a human TM. This model offers a controlled environment for testing the functionality and interactions between the SRV-coated tube and the surrounding tissue layers. By developing this novel model, we aim to create a method for studying the behavior of SRV-coated TTs in vitro, providing insights that could inform the design and application of TTs in other models and further in clinical settings.

### 2.7. Kinetics Studies

#### 2.7.1. Kinetic Diffusion Sensitivity Test

Using our TM model, we inserted SRV-CIPRO- or SRV-placebo-coated tubes daily in the bilayer model, in which the Tryptic Soy Agar was pre-seeded with *P. aeruginosa* and then incubated at 37 °C for 24 h [27], simulating conditions that mimic middle ear infections. The antibacterial efficacy was measured by determining the clearance zone, recorded as a cleared area (cm^2^), after incubation. Next, tubes were aseptically transferred to a new TM model for further incubation, and the zone of inhibition surrounding the tubes was measured daily.

#### 2.7.2. Effect on Planktonic Bacterial Growth

TTs were incubated daily in 3 mL of TSBG medium with 30 µL of an overnight bacterial culture for 24 h. The optical density (OD) at 600 nm was measured, and the bacterial concentration in the supernatant–fluid was determined. Control experiments involved TSBG incubated with the tubes in the absence of bacteria. The turbidity induced by CIPRO in the medium was considered.

#### 2.7.3. Biofilm Metabolic Assay

The metabolic activity of biofilms was measured daily using the MTT method [28]. The biofilms were exposed to 0.5 mg/mL MTT (3-(4,5-dimethyl thiazol-2-yl)-2,5-diphenyltetrazolium bromide) (Sigma, St. Louis, MO, USA) for 1 h, after which the tetrazolium crystals formed were dissolved in DMSO. The absorbance was measured at OD_570_ nm to quantify metabolic activity.

#### 2.7.4. BacTiter-Glo^TM^ Microbial Cell Viability Assay

The BacTiter-Glo^TM^ microbial cell viability assay was used to determine the number of viable bacterial cells based on ATP (Adenosine triphosphate) quantification. Each 100 µL sample was mixed with 100 µL of the BacTiter-Glo^TM^ reagent (Promega, Madison, WI, USA) in 96-well plates. After mixing for 5 min on an orbital shaker, luminescence was measured using a Tecan M200 plate reader [29].

#### 2.7.5. High-Resolution Scanning Electron Microscope (HR-SEM)

Biofilm formation on TT samples was prepared as described above. The biofilms were washed with phosphate-buffered saline (PBS) and twice with distilled deionized water (DDW) before being fixed with 4% paraformaldehyde for 20 min. After fixation, the samples were washed with DDW, dried, coated with iridium, and visualized using a MagellanTM 400L High-Resolution Scanning Electron Microscope (FEI Company, Hillsboro, OR, USA), with magnifications ranging from 200× to 20,000× [26].

#### 2.7.6. Spinning Disk Confocal Laser Scanning Microscopy (SDCLSM)

Biofilms were prepared as described above. After rinsing, the biofilms were stained with SYTO 9 and propidium iodide (PI) fluorescent dyes (Life Technologies, Carlsbad, CA, USA) for 30 min in the dark [30]. Stained samples were washed with PBS and visualized using a Nikon Yokogawa W1 Spinning Disk microscope with 50 µm pinholes. Fluorescence signals were recorded using a 10×/0.45 lens (Plan-Apochromat Lambda) and a SCMOS ZYLA camera. SYTO 9 (live cells) was detected at 488 nm excitation/515 nm emission, while PI (dead cells) was measured at 543 nm excitation/570 nm emission. Biofilm depth was analyzed with optical sections captured at 2.5 µm intervals. Viable *P. aeruginosa* cells in the biofilm were quantified via fluorescence intensity using NIS-element software (NIH, Bethesda, MD, USA). Data were expressed as the viable cells per biofilm layer and percentage of viable cells in the total biomass. Results were compared between SRV-CIPRO and SRV-placebo groups under identical conditions.

### 2.8. Statistical Analysis

The statistical mean of three or four independent experiments was calculated (for each experiment we evaluated at least 3 TTs with placebo and 3 TTs with SRV-CIPRO). Statistical significance was assessed using the Student’s *t*-test, with a *p*-value of less than 0.05 considered statistically significant.

## 3. Results

### 3.1. The Agar Disk Diffusion Assay Demonstrates the Extended Antibacterial Activity of SRV-CIPRO

**Figure 3:**
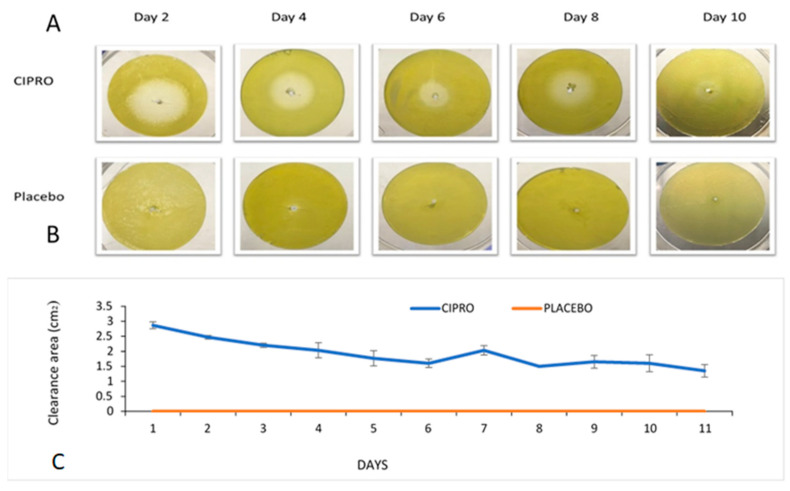
(**A**) Bacterial clearance around SRV-CIPRO-coated tubes plated on fresh *P. aeruginosa* agar plates. (**B**) The SRV-placebo-coated tubes showed no clearance of bacteria. (**C**) The average bacterial inhibition zones observed after daily transfer of SRV-CIPRO- and SRV-placebo-coated tubes on fresh *P. aeruginosa* agar plates.

Using our TM model, the results demonstrated that SRV-CIPRO-coated TTs effectively inhibited *P. aeruginosa* growth for at least 10 ± 1 days (Figure 3A). In contrast, SRV-placebo-coated tubes showed no antibacterial activity (Figure 3B). During the first seven days, the clearance zone was at its maximum size, indicating a strong initial antibacterial effect. Even at later time points, a sustained release of CIPRO maintained bacterial inhibition. Some variability in the clearance zone size was observed throughout the experiments, likely due to fluctuations in CIPRO release rates. These findings highlight the potential clinical application of sustained-release TTs in preventing middle ear infections.

### 3.2. Antibacterial Impact of the Coated Tube on Planktonic Bacterial Proliferation

**Figure 4:**
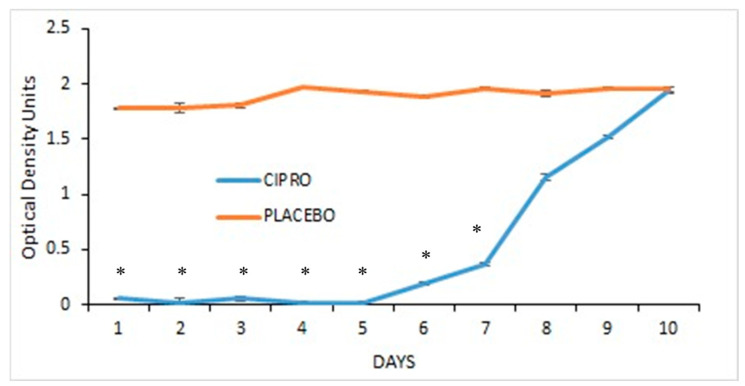
SRV-CIPRO- and SRV-placebo-coated tubes were incubated daily with planktonic-growing *P. aeruginosa*, and the OD_600_ was measured after 24 h. * *p* < 0.05 for SRV-CIPRO-coated tubes versus SRV-placebo-coated tubes.

TTs coated with SRV-CIPRO showed a significant reduction in *P. aeruginosa* growth compared to SRV-placebo-coated tubes, with a marked effect from day 1 through day 5, after which bacterial growth began to gradually increase until day 10, where there was no further inhibition (Figure 4).

### 3.3. Biofilm Metabolic Assay

**Figure 5:**
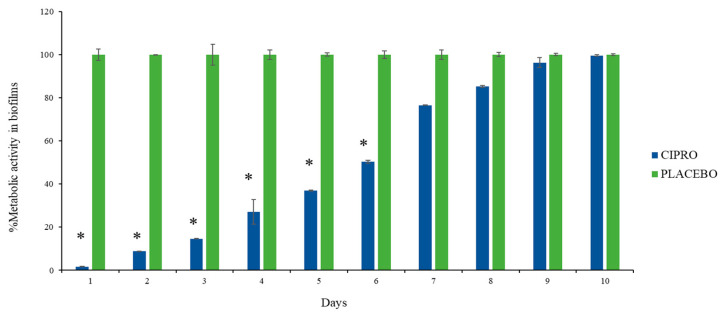
SRV-CIPRO-coated tubes effectively and significantly reduce biofilm metabolic activity during the first 6 days of exposure to *P. aeruginosa*, as determined by the MTT metabolic assay. *n* = 3, * *p*-value < 0.05.

The biofilm metabolic assay showed a significant reduction in bacterial metabolism up to day 6 in the medium exposed to SRV-CIPRO-coated tubes compared to SRV-placebo-coated tubes (Figure 5). After day 6, the SRV-CIPRO tubes showed a minor reduction that was recorded until day 10. This finding suggests that the SRV-CIPRO coating effectively reduced bacterial metabolic activity over time, resulting in less biofilm accumulation (Figure 5). The sustained release of ciprofloxacin from the SRV-CIPRO-coated tubes provided continuous antibiotic exposure to the surrounding environment, which likely interfered with the early stages of biofilm development, thereby preventing bacteria from forming mature biofilms. In contrast, the SRV-placebo-coated tubes, which lacked an active antibacterial agent, allowed biofilm metabolic activity to progress, leading to constant and higher levels of bacterial metabolism in the biofilm.

### 3.4. BacTiter-Glo^TM^ Microbial Cell Viability Assay

**Figure 6:**
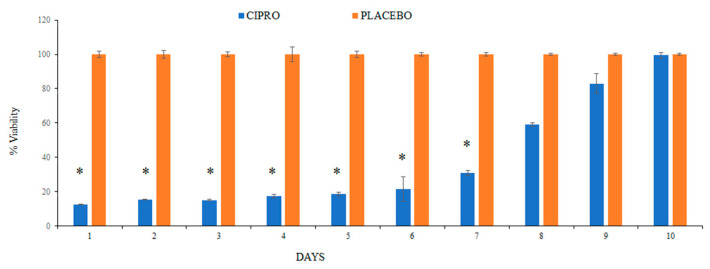
The SRV-CIPRO coating demonstrated a statistically significant reduction in the number of viable *P. aeruginosa* over a 7-day period compared to the SRV-placebo coating, as determined by the BacTiter-Glo^TM^ microbial cell viability assay. * *p*-value < 0.05.

To further assess the viability of the bacterial cells, we measured ATP levels, which serve as a reliable indicator of cell viability. ATP, the molecule responsible for storing and transferring energy within cells, is only produced by living cells, making it an effective marker for bacterial activity and survival. The results revealed a significantly lower number of viable bacteria in the bacterial culture exposed to SRV-CIPRO-coated tubes compared to those exposed to SRV-placebo-coated tubes. This reduction in bacterial viability persisted consistently and significantly throughout the first 7 days, indicating that the sustained release of ciprofloxacin from the coated tubes effectively controlled bacterial viability during this period (Figure 6). The continuous release of the antibiotic likely disrupted key bacterial processes, limiting proliferation. However, after day 7, bacterial viability in the SRV-CIPRO-treated group gradually began to increase. By day 10, the number of viable bacteria had risen to levels comparable to those observed in the SRV-placebo group. This increase suggests that the concentration of ciprofloxacin released after day 7 may have diminished enough to allow bacterial growth to resume and eventually match the placebo-treated samples. These findings illustrate both the initial efficacy of the SRV-CIPRO coating in controlling bacterial growth and the need for monitoring its long-term effectiveness.

### 3.5. High-Resolution-Scanning Electron Microscope (HR-SEM)

**Figure 7:**
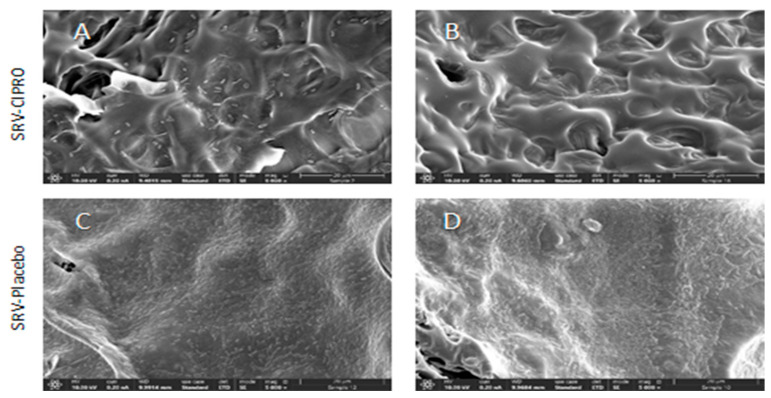
HR-SEM images of SRV-CIPRO-coated (**A**,**B**) and SRV-placebo-coated (**C**,**D**) tube surfaces that have been exposed seven times to planktonic-growing *P. aeruginosa*. The ×5000 magnification is shown.

HR-SEM images revealed a considerable inhibition of biofilm formation on the TTs coated with SRV-CIPRO for *P. aeruginosa* at day 7 compared to the SRV-placebo-coated tubes (Figure 7). The presence of biofilms of *P. aeruginosa* were evident on the SRV-placebo-coated tubes (Figure 7C,D), while only scattered clusters of the bacteria were observed on the SRV-CIPRO-coated tubes (Figure 7A,B). These findings indicate that coating the tubes with SRV-CIPRO significantly inhibits biofilm formation by *P. aeruginosa*.

### 3.6. Spinning Disk Confocal Laser Scanning Microscopy (SDCLSM)

**Figure 8:**
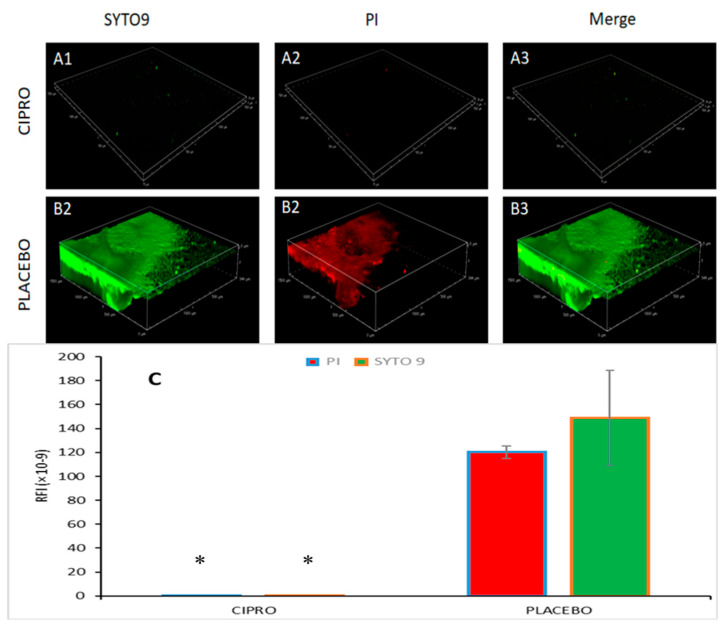
SDCLSM images of SYTO 9/PI staining of *P. aeruginosa* biofilms formed on SRV-CIPRO- (**A1**–**A3**) and SRV-placebo- (**B1**–**B3**) coated tubes after 3 days of exposure to bacteria. The tubes were visualized using a Nikon Yokogawa W1 Spinning Disk Confocal microscope with a 10×/0.45 Plan-Apochromat Lambda objective. (**C**) Quantification of the stained biofilms coated with either SRV-CIPRO or SRV-placebo. * *p* < 0.05. The images cover a biofilm area of 1500 µm × 1500 µm.

Spinning disk confocal laser scanning microscopy (SDCLSM) images revealed that SRV-CIPRO-coated tubes, following three days of exposure to planktonic *P. aeruginosa*, exhibited only weak green fluorescence emission (live bacteria) with minimal red fluorescence emission (dead bacteria) (Figure 8(A1–A3)). In contrast, SRV-placebo samples were largely covered by a biofilm predominantly composed of live bacteria (Figure 8(B1–B3)), indicating that the SRV-placebo coating did not significantly reduce overall bacterial colonization. Quantitative analysis of the green and red fluorescence intensities confirmed that both live and dead bacterial populations were markedly lower on the SRV-CIPRO-coated tubes (Figure 8C). These results demonstrate that the tubes coated with SRV-CIPRO had considerably less bacterial colonization compared to the SRV-placebo, further emphasizing the antibacterial efficacy of SRV-CIPRO in reducing biofilm formation.

## 4. Discussion

The objective of this study was to develop and assess a novel approach utilizing a local sustained-release technology, incorporated with ciprofloxacin, to prevent and manage early postoperative complications, particularly otorrhea due to infection, following TT insertion. TT insertion is widely used for the management of chronic OME and recurrent AOM [5]. Although highly effective, this procedure is often associated with complications such as early and late ear discharges, which pose challenges for both clinicians and patients [19].

Our study demonstrated that the SRV-CIPRO-coated TTs might reduce the incidence of early postoperative otorrhea. This finding highlights the potential of sustained-release local antibiotic delivery technology as a superior alternative to conventional postoperative care methods. These include advantages such as reducing the repeated administration of topical ciprofloxacin, as in ear drops, which is an important patient compliance issue, especially in younger populations. The localized, continuous release of ciprofloxacin from the varnish ensures that therapeutic concentrations of the antibiotic are maintained at the site of potential infection for a prolonged period of time. This outcome aligns with previous studies that have underscored the effectiveness of ciprofloxacin in managing otorrhea when used as ear drops [13,14]. However, traditional methods require multiple applications, presenting challenges for patient compliance, particularly in pediatric cases. By incorporating ciprofloxacin into a sustained-release system, we have addressed the need for a more efficient delivery mechanism that eliminates the difficulties associated with conventional administration.

Previous studies have demonstrated that TTs reduce the recurrence of AOM and improve hearing outcomes in the short to medium term [31]. However, complications such as otorrhea remain a persistent issue. In comparison to the standard use of ciprofloxacin ear drops, our novel SRV-CIPRO-coated tubes offer enhanced protection against these complications by ensuring continuous drug release at the site of the TT, which bears a strong pharmacological advantage.

In this study, we have developed a simplistic initial model of insertion of a TT into an environment that may resemble the TM environment.

The model consisted of two layers: a transparent plastic roll to replicate the TM’s outer flexibility and translucency zone, and a thin and more gelatinous layer of Tryptic Soy Agar (TSA). A small incision allowed for the insertion of the TT, simulating its positioning in the TM. This controlled in vitro model enabled us to assess the tube’s functionality and interaction with tissue-like layers. The effect of applying forces on the coated TT, the release of the agent in a gelatinous environment, and the duration of release can be assessed in this model. These conditions provide valuable insights into the behavior of TTs and the efficacy of SRV coatings in preventing infections, such as otorrhea. The model we used is a step further towards cell/bacteria models and in vivo clinical trials, as it provides better simulation of the conditions relevant to clinical outcomes, allowing for a more realistic assessment of TT performance in a setting similar to the human middle ear environment.

The study by Roland et al. [19] demonstrated that ciprofloxacin ear drops were effective in managing postoperative otorrhea. Our hypothesis is based on this research by showing that sustained release of ciprofloxacin via the SRV coating could not only match the effectiveness of ear drops but potentially surpass it by ensuring better patient compliance and a reduced risk of infection without requiring caregiver intervention in the early postoperative period. The pharmaceutical technology of local sustained-release delivery systems has seen remarkable progress in recent years. Various pharmaceutical applications have been developed for dental treatments, cancer therapy, and wound healing.

Several coating procedures have been suggested in the ear–nose–throat clinical field.

Coating of stents has been suggested in the literature as a therapeutic means to reduce biofilms on tympanostomy stents. TiO_2_ has reduced biofilm formation of *P. aeruginosa* [32].

Polyvinylpyrrolidone (PVP), silver oxide or silver, or combination coatings of tympanostomy tubes reduced *P. aeruginosa* biofilm formation, while PVP was superior to silver and PVP-silver [33]. However, PVP and PVP-silver coatings nominally increased *S. aureus* biofilm formation. Additionally, TTs coated with fluoroplastic reduced biofilm formation [34].

It is feasible that a technology of sustained release of the agents would probably enhance the efficacy of the suggested coatings and increase patient and caregiver compliance to the treatment.

The pharmacological concept of a sustained-release delivery system is now being further explored for ear, nose, and throat applications, including a sinonasal stent and voice prosthesis coated with a SRV containing chlorhexidine [35,36]. Using SRV to coat various ENT devices that are prone to biofilm formation is a novel approach that may positively impact post-surgical outcomes [35]. Furthermore, this pharmaceutical drug delivery system could serve as an innovative platform in otolaryngology for the localized and prolonged duration of application of other antibacterial, antifungal, or anti-inflammatory agents. Moreover, the emergence of bacteria which are ciprofloxacin-resistant is recognized worldwide. Few suggestions other than new drugs or drug combinations have been offered in the literature in order to overcome this world-wide clinical issue [37,38,39]. An SRV containing a drug or multiple drugs that bacteria are sensitive to may be a new avenue for minimizing infections due to the presence of resistant bacteria.

This is a feasibility study on developing an innovative means to affect microbial associated pathogenic conditions in otolaryngology using a sustained-release technology. Further studies on different bacterial strains, such as *Staphylococcus aureus*, a heterogeneous bacterial model, and an improved tympanic membrane model should follow before embarking on animal and human studies. Investigating the biological release kinetics is a preliminary step in validation of the developed technology. The pharmacological release kinetics will add more substantial information as to the properties of the new concept. On top of the pharmacological advantages and improved patient compliance, this new technology needs to be compared to a traditional treatment in postoperative complications associated with TT insertion.

## 5. Conclusions

This study presents the development of a new approach for managing postoperative complications associated with TTs insertion by utilizing a local coating of SRV containing ciprofloxacin. The prolonged duration of the antibacterial/antibiofilm effects of the TT coated with SRV, which lasted for 10 days, offers a promising solution for reducing early postoperative complications, improving patient compliance, and minimizing the risks associated with systemic antibiotics. By addressing both clinical and practical challenges, this innovation has the potential to enhance patient outcomes and redefine postoperative care in otolaryngology.

## Data Availability

The original contributions presented in this study are included in the article. Further inquiries can be directed to the corresponding author.
